# A new species of *Boholina* (Crustacea, Copepoda, Calanoida) and a first record for stygobiotic calanoid fauna from a cave in Thailand

**DOI:** 10.3897/zookeys.904.37609

**Published:** 2020-01-16

**Authors:** Chaichat Boonyanusith, Koraon Wongkamhaeng, Sujeephon Athibai

**Affiliations:** 1 School of Biology, Faculty of Science and Technology, Nakhon Ratchasima Rajabhat University, Nakhon Ratchasima 30000, Thailand Nakhon Ratchasima Rajabhat University Nakhon Ratchasima Thailand; 2 Department of Zoology, Faculty of Science, Kasetsart University, Bangkok 10900, Thailand Kasetsart University Bangkok Thailand; 3 Applied Taxonomic Research Center and Department of Biology, Faculty of Science, Khon Kaen University, Khon Kaen 40002, Thailand Khon Kaen University Khon Kaen Thailand

**Keywords:** anchialine cave, cave-dwelling copepod, Pseudocyclopidae, Satun Province, Southeast Asia

## Abstract

A new species of Calanoida belonging to the genus *Boholina* Fosshagen & Iliffe, 1989 was found in a freshwater pool within a cave of the Satun province, South Thailand. It is the first record of the genus and of a stygobiotic representative of calanoid fauna in this country. The new species is most similar to *B. crassicephala* Fosshagen & Iliffe, 1989, based on position of genital pores, structures of P4 and P5 in both sexes, relative length of subapical spine vestige on the male right P5, and shape of the male left P5 endopods. However, this new species is distinguished from its known congeners by: (1) relatively longer distal outer spines on the male right P5 exopods, (2) smaller endopods of the male left P5 and (3) elongated apical spines on the distal exopodal segment of the female P4 and P5. Furthermore, the distinctive characteristic of the Thai *Boholina* is the presence of inner minute seta on the distal segment of the male right P5 exopod. Detailed descriptions of the new species and a key to all six known species of the genus *Boholina* is provided.

## Introduction

The study of the Copepoda diversity in Southeast Asia is progressing rapidly due to an intensive program coordinated by Prof. La-orsri Sanoamuang and her colleagues. During 12 years of intensive sampling of cave-dwelling copepods in Thailand and Vietnam, many new Cyclopoida and Harpacticoida have been presented to science (Brancelj et al. 2010; Watiroyram et al. 2012, 2015a, 2015b, 2017; Boonyanusith et al. 2013, 2018a, 2018b; Watiroyram and Brancelj 2016; Karanovic et al. 2017; Watiroyram 2018a, 2018b; Sanoamuang et al. 2019). Among Cyclopoida, two endemic genera from Thailand and Vietnam were established, including *Siamcyclops* Boonyanusith, Sanoamuang & Brancelj, 2018, and *Pseudograeteriella* Sanoamuang, Boonyanusith & Brancelj, 2019 (Boonyanusith et al. 2018b; Sanoamuang et al. 2019).

Various calanoid copepods showing plesiomorphic features, belonging to the families Boholinidae, Ridgewayiidae (now both synonyms of the family Pseudocyclopidae) and Epacteriscidae were discovered from several anchialine environments in tropical and subtropical waters around the world (e.g., Fosshagen and Iliffe 1989, 1991, 2004a, 2004b; Fosshagen et al. 2001; Jaume and Humphreys 2001; Boxshall and Jaume 2003, 2012; Suárez-Morales and Iliffe 2007; Figueroa 2011; Moon and Soh 2014). However, stygobiotic Calanoida have never been recorded in Thailand to date.

During the investigation of cave-dwelling Copepoda in the Satun province, South Thailand, a representative of Calanoida was collected from a freshwater pool within a cave. Based on the unique characteristic of a grasping organ on the males’ left P5, the cuticular pointed projection on the caudal rami (Fosshagen and Iliffe 1989), and the presence of an additional element on the inner margin of the males’ right P5 exopod, a new species of the genus *Boholina* Fosshagen & Iliffe, 1989 was identified representing the first record of the genus and of the stygobiotic representative of calanoid species in Thailand.

**Figure 1.**
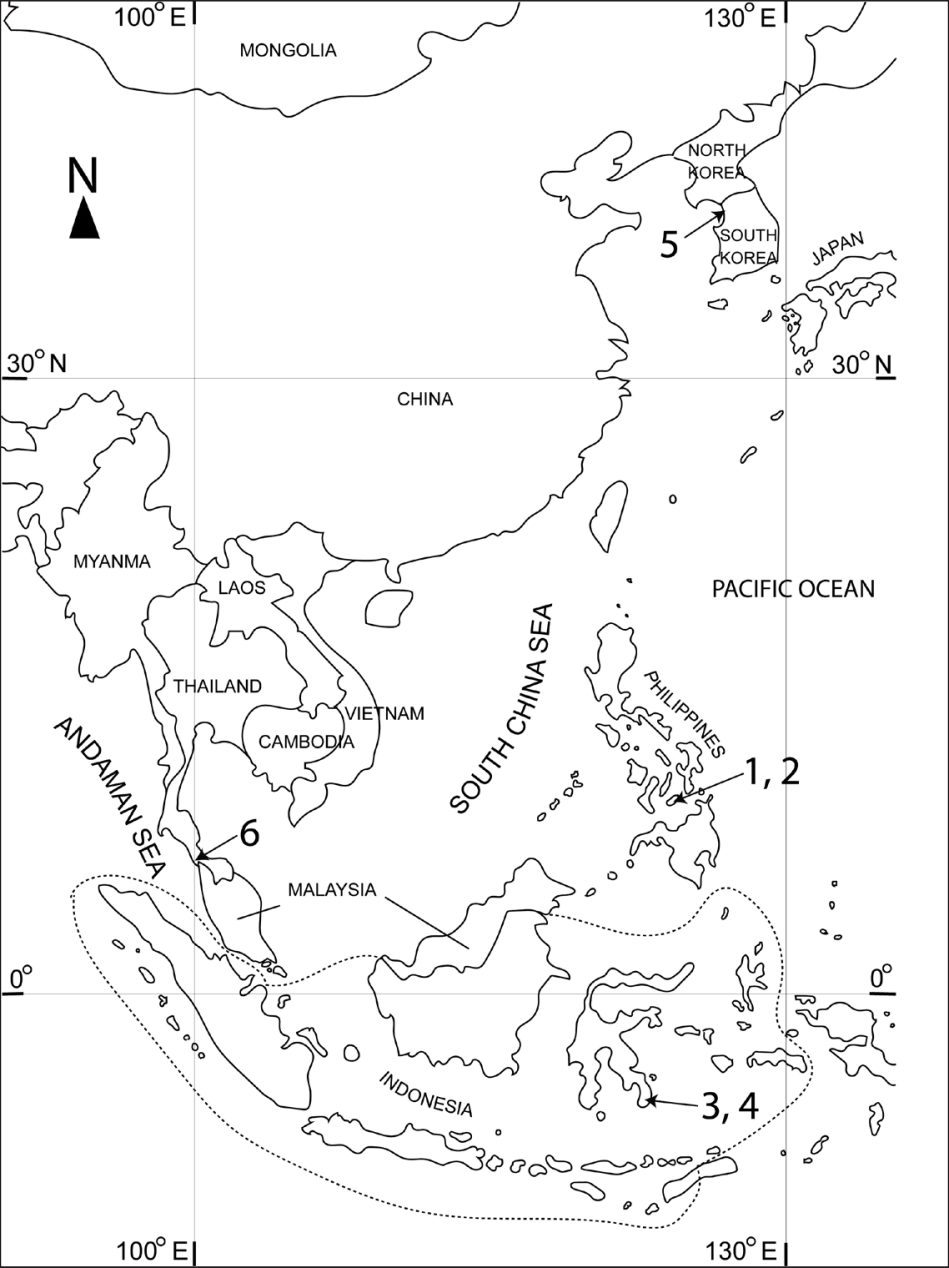
Distribution of the representatives of the genus *Boholina*: **1***B. crassicephala***2***B. purgata***3***B. parapurgata***4***B. munaensis***5***B. ganghwaensis***6***B. laorsriae* sp. nov.

According to Fosshagen and Iliffe (1989), the family Boholinidae and the genus *Boholina* were established on the basis of two new species collected from a brackish pool in San Vicente cave on Bohol Island in the Philippines. To date, five species are recognised in this genus, only known from East and Southeast Asia (Fig. 1); they are *B. crassicephala* Fosshagen & Iliffe, 1989, and *B. purgata* Fosshagen & Iliffe, 1989, recorded from a pool in the San Vicente Cave on Bohol Island in the Philippines, *B. munaensis* Boxshall & Jaume, 2012 from a spring in Lawou Cave on Muna Island in Indonesia, *B. parapurgata* Boxshall & Jaume, 2012 from sinkholes on the coast of Muna Island, Indonesia, and *B. ganghwaensis* Moon & Soh, 2014 from the burrows of *Cleistostoma dilatatum* (De Haan, 1833) in the inter-tidal mudflat of Ganghwa Island, western Korea (Fosshagen and Iliffe 1989; Boxshall and Jaume 2012; Moon and Soh 2014). More recently, the families Boholinidae and Ridgewayiidae were synonymised in the family Pseudocyclopidae by the morphology-based phylogenetic work of Bradford-Grieve et al. (2014).

In Thailand, six specimens of *Boholina* were collected from a freshwater pool within a cave. This type locality is in an area that has recently been designated as a UNESCO global geopark.

## Materials and methods

**Figure 2.**
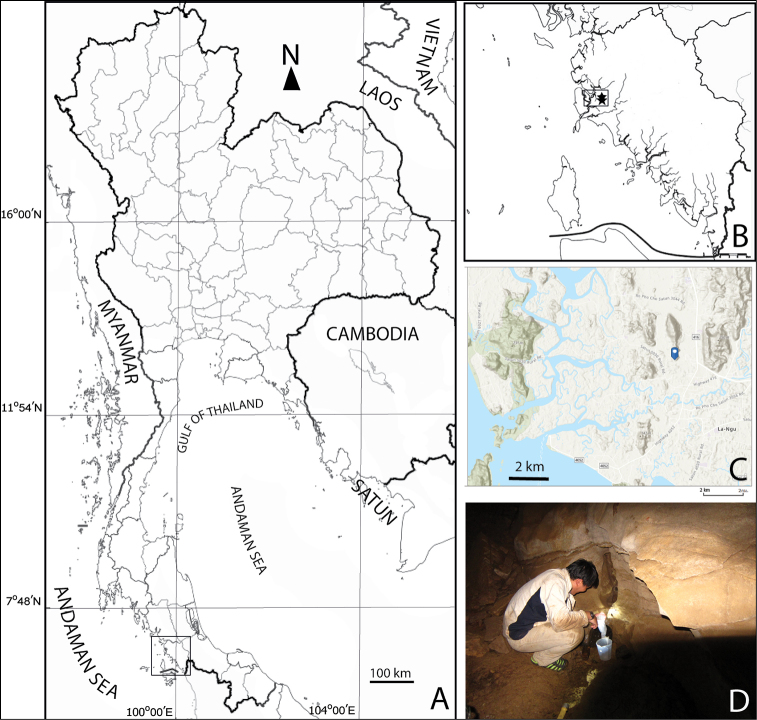
Geographical location and details of sampling site: **A** map of Thailand and location of Satun province **B** sampling location of cave in Satun province (indicated by a star) **C** topography of area around the hill in which the cave is located **D** sampling point in cave.

Samples were collected from a pool in Khay Cave of Satun province, South Thailand (Fig. 2) by hand net with a mesh size of 60 µm. They were placed in a plastic bottle with a 4 % formaldehyde solution as a fixative. In the laboratory, specimens were sorted under a stereomicroscope and stored in 70 % ethanol. They were placed in a mixture of glycerol and 70 % ethanol (ratio ~1:10 v/v) for 30 minutes before the morphological examination. Examination of habitus was done on the male and female specimens, which were placed in a drop of glycerol between a pair of coverslips on slide. Specimens were then dissected and mounted on slides using glycerol as a mounting medium. The examination was made with a Nikon ECLIPSE E200 compound light microscope at a magnification of ×1000. Habitus and dissected body parts were drawn using a drawing tube attached to a compound microscope, and the final versions of illustrations were prepared by Adobe Illustrator CC 2017. Description follows Huys and Boxshall (1991). The following descriptive abbreviations are used throughout the text and figures:

**Endp** endopod;

**Exp** exopod;

**Endp/Exp-1 (2, 3)** proximal (middle, distal) segment of rami;

**ae** aesthetasc;

**I** spine;

**P1–P5** swimming legs 1–5.

Type material has been deposited at the Princess Maha Chakri Sirindhorn National History Museum, Prince of Songkla University, Songkhla, Thailand (**PSUNHM**).

## Taxonomy

### Order Calanoida G.O. Sars, 1903

#### Family Pseudocyclopidae Giesbrecht, 1893

##### Genus *Boholina* Fosshagen & Iliffe, 1989

###### Boholina laorsriae sp. nov.

Taxon classificationAnimaliaCalanoidaPseudocyclopidae

4B924358-2ACB-5D6C-B87B-806B21DA4BA5

http://zoobank.org/E1E3D8D5-7B4E-4DD8-86F8-AAC296A21649

**Figure 3.**
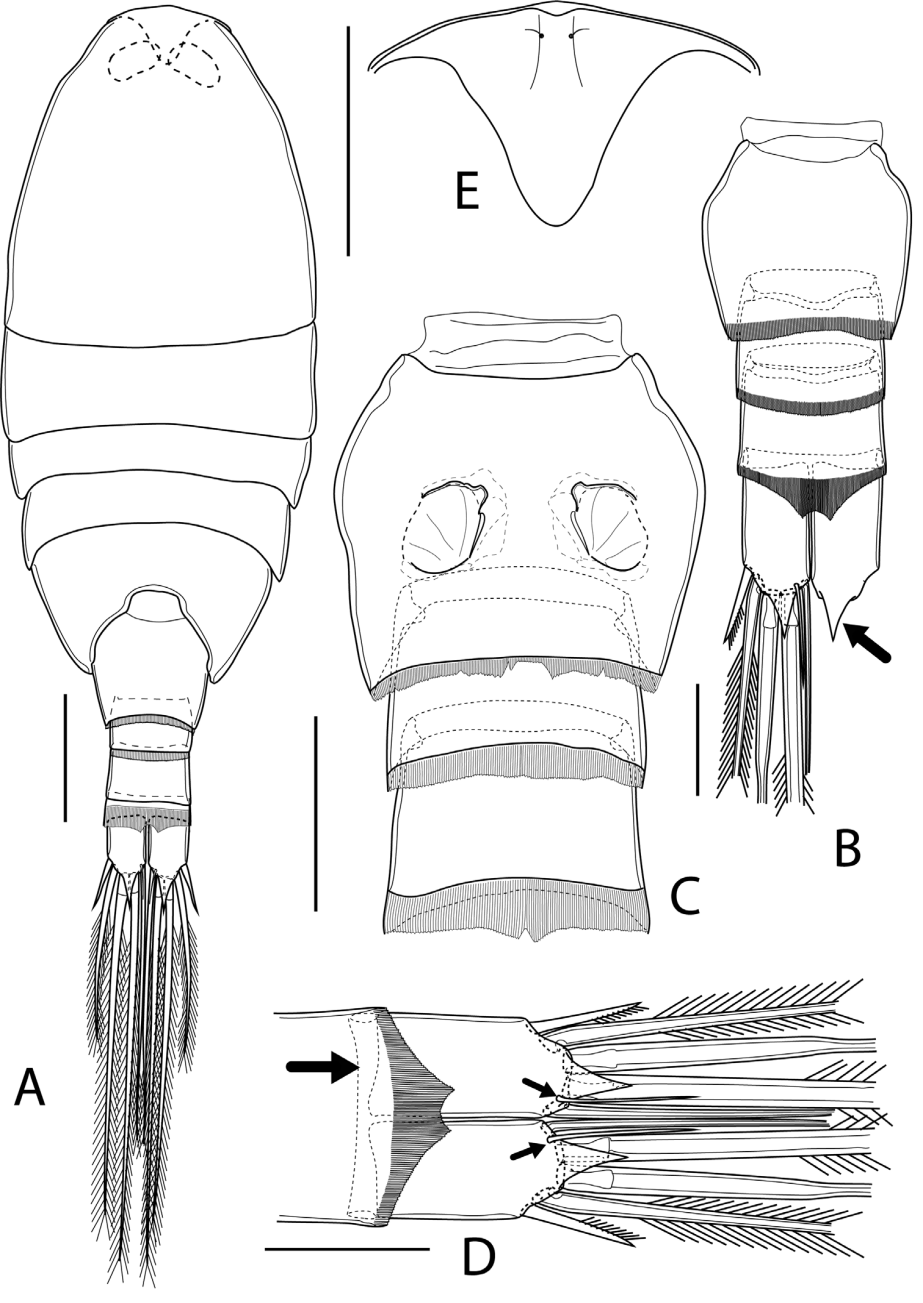
*Boholina laorsriae* sp. nov. female: **A** habitus, dorsal view **B** urosome, dorsal view **C** genital double-somite, ventral view **D** caudal rami, dorsal view **E** rostrum, frontal view. Scale bars: 100 μm (**A**); 50 μm (**B−E**).

**Figure 4.**
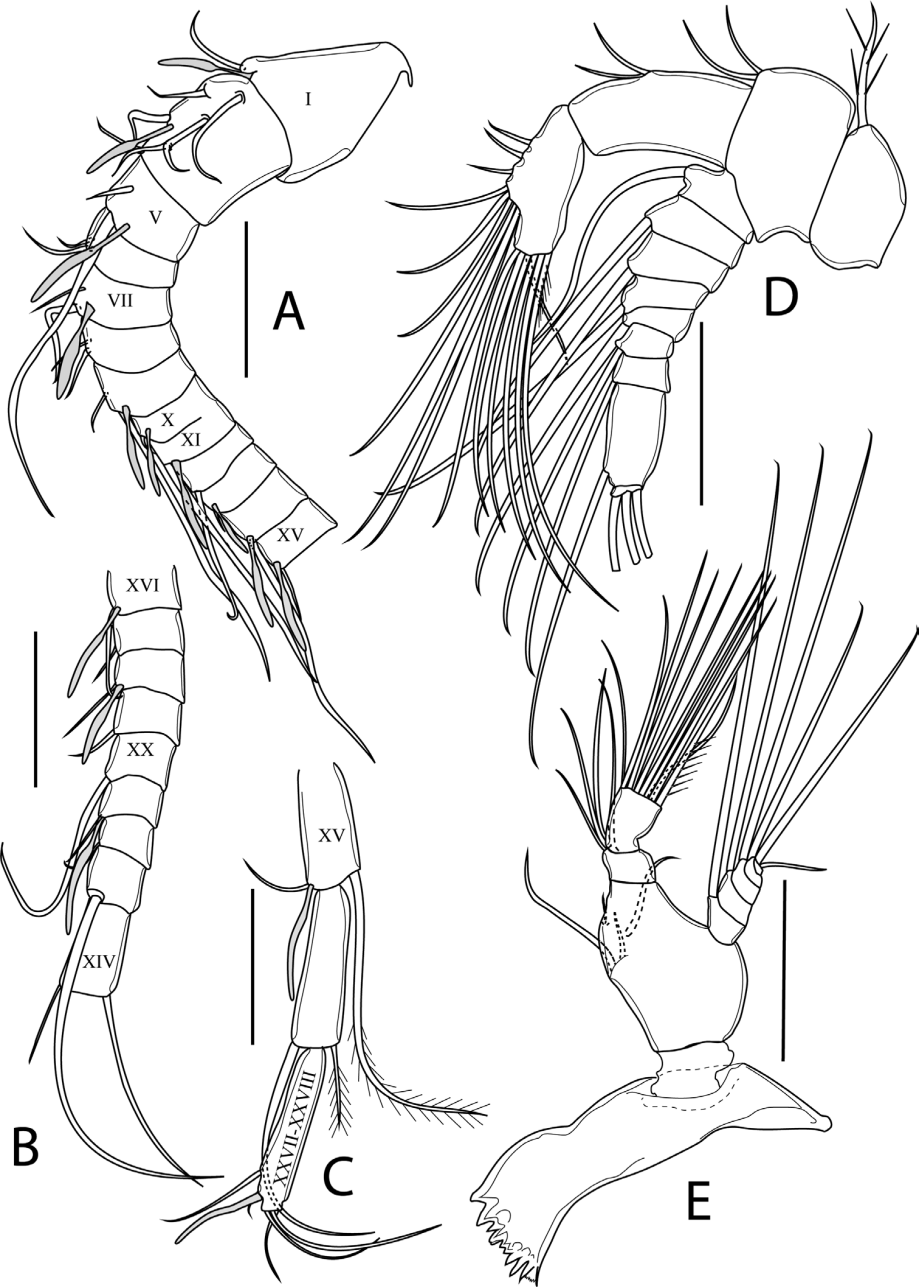
*Boholina laorsriae* sp. nov. female: **A** segments 1−12 of antennule **B** segments 13−21 of antennule **C** segments 22−24 of antennule **D** antenna **E** mandible. Scale bars: 50 μm. Roman numerals on antennule correspond to ancestral segments.

**Figure 5.**
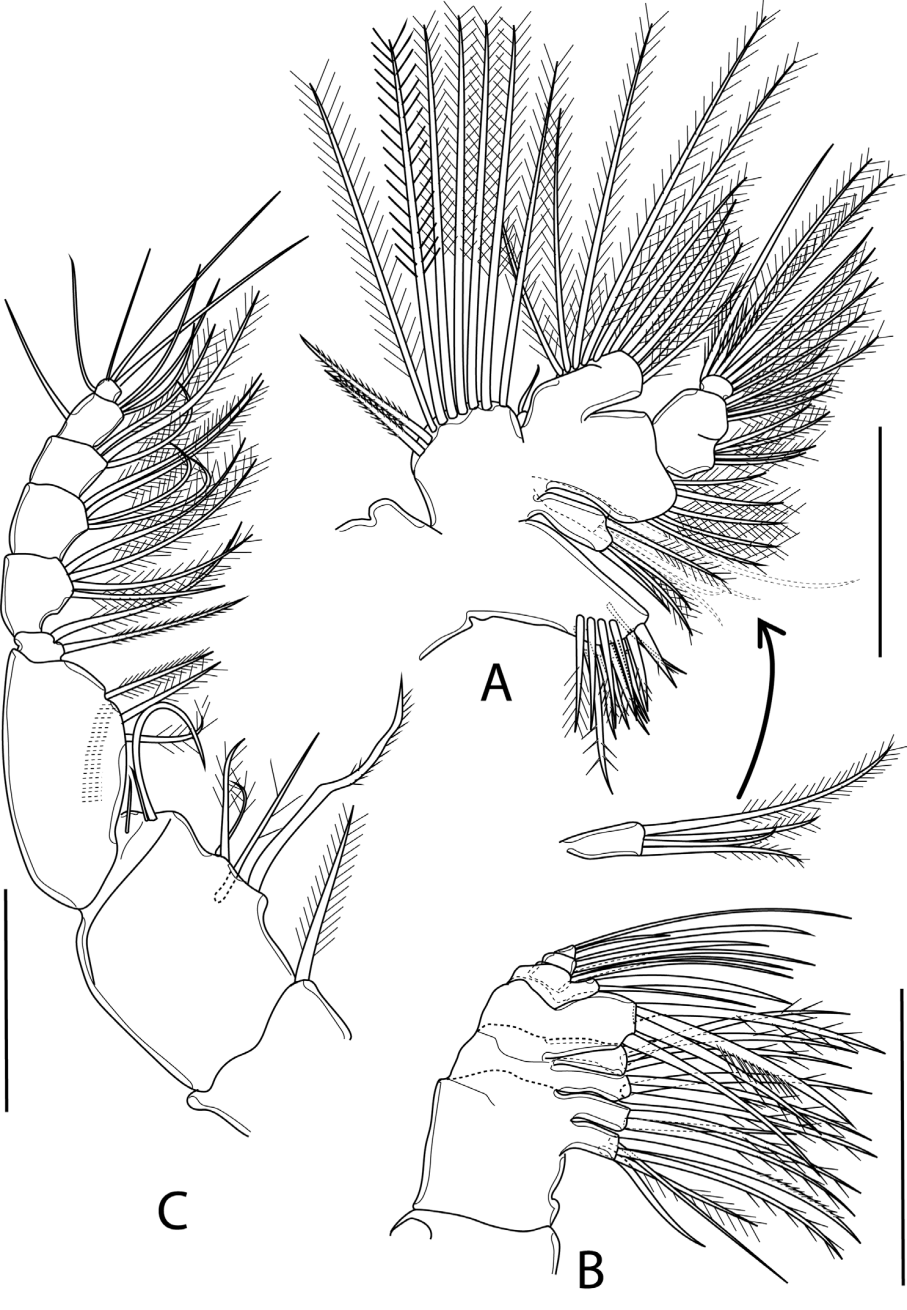
*Boholina laorsriae* sp. nov. female: **A** maxillule **B** maxilla **C** maxilliped. Scale bars: 50 μm.

**Figure 6.**
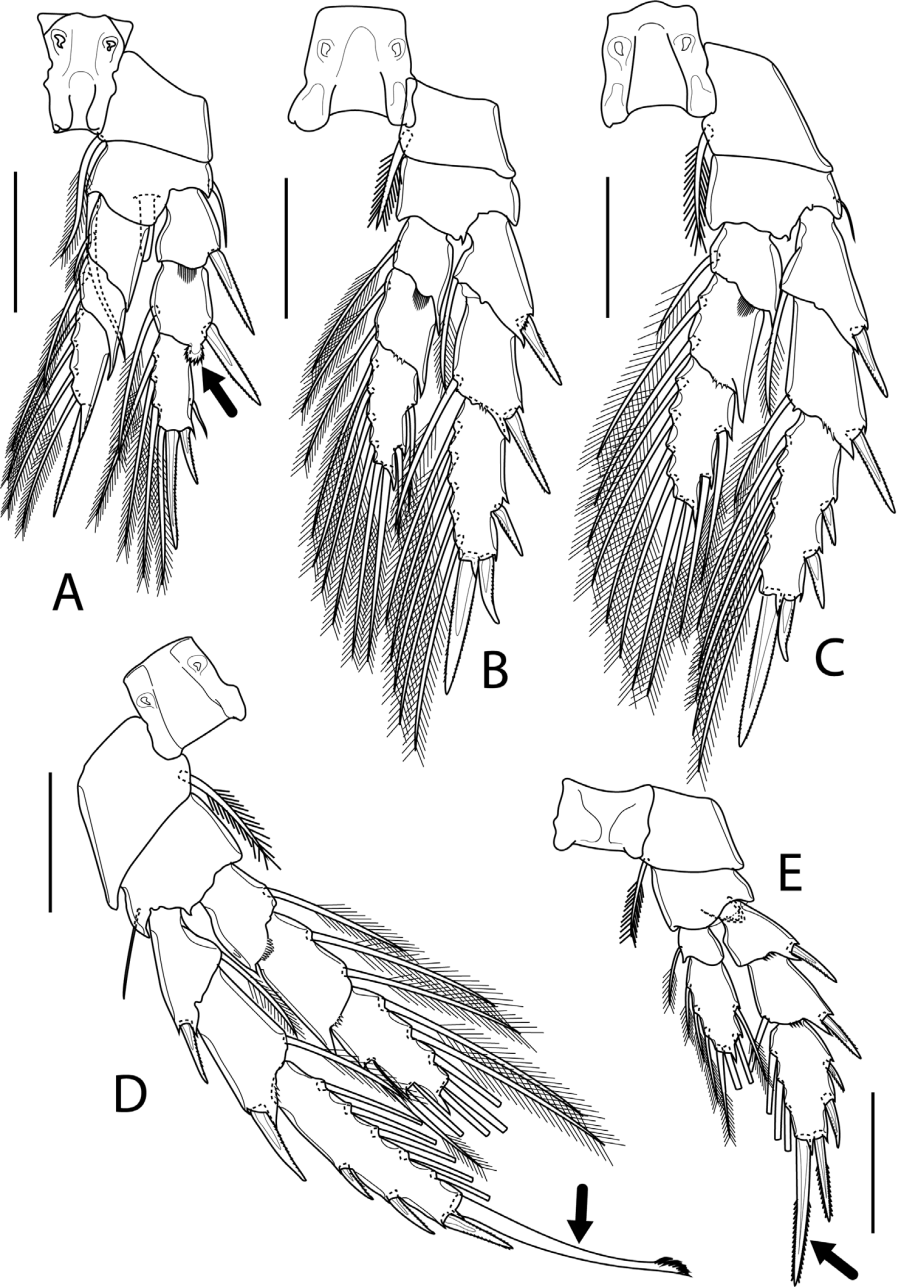
*Boholina laorsriae* sp. nov. female: **A**P1**B**P2**C**P3**D**P4**E**P5. Scale bars: 50 μm.

**Figure 7.**
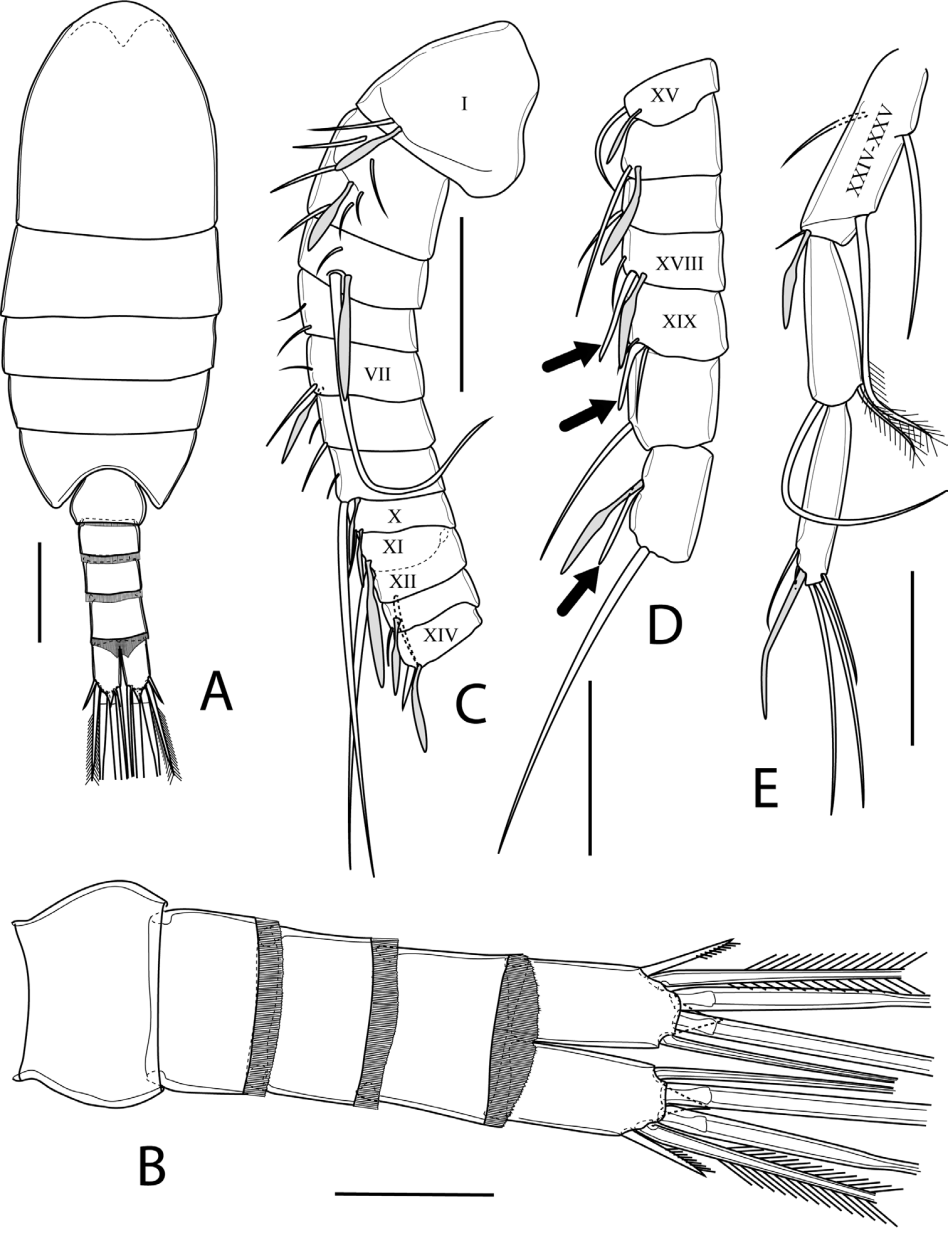
*Boholina laorsriae* sp. nov. male: **A** habitus, dorsal view **B**: urosome, ventral view **C** segments 1−12 of antennule **D** segments 13−19 of antennule **E** segments 20−22 of antennule. Scale bars: 100 μm (**A**); 50 μm (**B−E**). Roman numerals on antennule correspond to ancestral segments.

**Figure 8.**
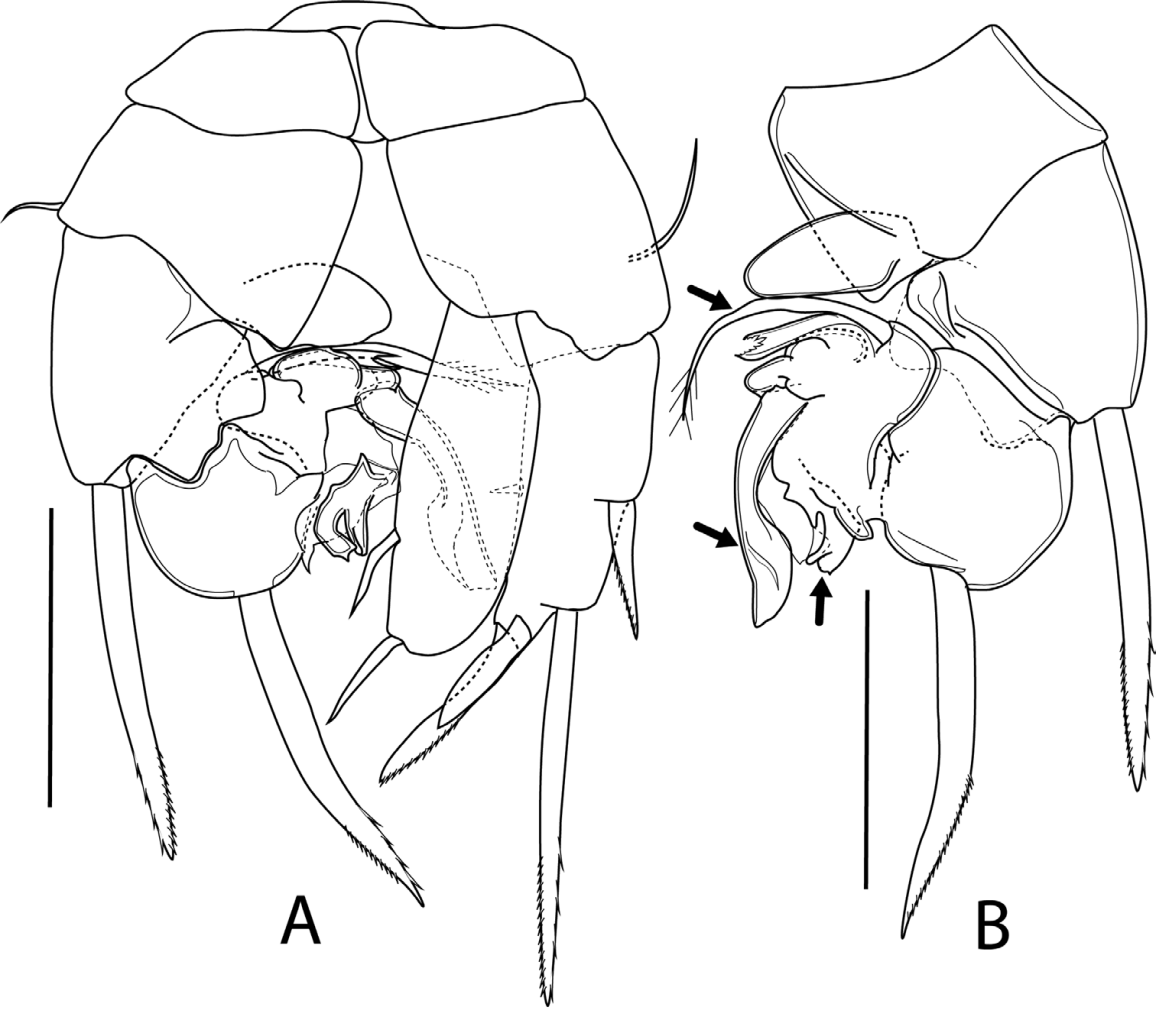
*Boholina laorsriae* sp. nov. male: **A**P5 caudal view **B** left rami of P5. Scale bars: 50 μm.

Figs 3 , 4 , 5 , 6 [female]; Figs 7 , 8 [male]

####### Material examined.

***Holotype***: THAILAND • ♀ (adult), 0.73 mm long; Satun Province, Khay Cave; 6°53'40"N, 99°46'44"E, 17 m a.s.l.; 17 December 2014; C. Boonyanusith leg.; hand net; completely dissected and mounted on two slides in glycerol and sealed with nail vanish; PSUZC-PK2004-01–02. ***Allotype***: THAILAND • ♂ (adult), 0.67 mm long, collection data as for holotype; PSUZC-PK2004-03. ***Paratypes***: THAILAND • 1 ♀ (adult) and 1 ♂ (adult); same data as for holotype; PSUZC-PK2004-04–05.

####### Additional material.

THAILAND • 2 ♂♂ (adult); same data as for holotype; preserved in 70% ethanol; retained in collection of the first author (CB).

####### Etymology.

The species is named after Prof. Dr. La-orsri Sanoamuang (Khon Kaen University) in honour of her great and invaluable contribution on the knowledge of the planktonic fauna in Thailand. The name of species is a feminine noun in genitive singular.

####### Type locality.

The Khay Cave is in La-Ngu district, Satun province, ca. 760 km south of Bangkok (Thailand) (Fig. 2A). The cave is in an isolated, limestone hill of the Nakhon Sri Thammarat Mountain range, at an elevation of 17 m a.s.l, ca. 6.5 km from the Andaman Sea, (Fig. 2A–C). The cave has two entrances. The first one is located ca. 3 m over the hill floor and the second is at the base of the hill. Beyond the entrance is a horizontal gallery, which is ca. 20 m high. Occasionally, the gallery is inundated by freshwater during the rainy season. There is no permanent route connecting water in the cave and the sea; however, ca. 40 years ago, the cave was probably inundated by the sea water during the rising up of the sea water level (personal communication). The type locality is a small pool hidden under the cave wall with a small opening (Fig. 2D). It is ca. 10 m far from the first entrance and is seasonally filled by rain. The water temperature was 24.6 °C, pH 8.93, conductivity 450 µS cm^-1^, DO 5.7 mg L^-1^, and salinity 0.2 ppt.

####### Diagnosis.

***Female***: Pseudocyclopidae. Fourth and fifth pedigerous somites completely fused. Postero-lateral corners of cephalosome and first three pedigerous somite rounded. Genital double-somite barrel-shaped, ornamented with hyaline membrane all around the posterior margin; hyaline membrane with large medial notch ventrally. Genital pores paired, located ventrolaterally. Hyaline membrane of preanal somite expanded dorso-medially to form trapezoidal double-pointed flap. Caudal ramus with triangular pointed projection on distal margin. Antennule relative short, not reaching beyond distal margin of prosome. Apical spine on female P4Exp-3 elongated, ca. 3 × as long as outer terminal spine. Apical spine on female P5Exp-3 ca. 1.8 × as long as outer terminal spine. ***Male***: The left P5Exp-3 highly transformed, bearing three irregular lobes; Endp oval-shaped, much shorter than right P5Endp, ca. 1.6 × as long as wide. The male right P5Exp with minute inner spiniform seta; distal outer spine elongated, ca. 3.4 × as long as proximal outer one and ca. 2.7 × as long as apical spine; subapical spine vestige ca. 0.7 × as long as apical spine.

####### Description of adult female.

Body (Fig. 3A) with a total length of 0.68 and 0.73 mm (measured from anterior margin of cephalosome to tip of projection of caudal rami, mean: 0.71 mm; *N* = 2). Prosome 5-segmented, elliptical, ca. 70 % of body length and 2.5 × as long as urosome, with greatest width at posterior end of first pedigerous somite; greatest width ca. 43 % of prosome length. Cephalosome and first three pedigerous somites free; postero-lateral corners rounded. Fourth and fifth pedigerous somites completely fused (Fig. 3A); postero-lateral corners rounded, symmetrical. Naupliar eye not discernible. Urosome 4-segmented, comprising genital double-somite, two free abdominal somites and very short anal somite (Fig. 3A–D). Genital double-somite barrel-shaped, ca. 45 % of urosome length, with greatest width at mid-length of double-somite, with hyaline membrane all around the posterior margin; hyaline membrane with large medial notch ventrally. Genital pores paired, located ventrolaterally (Fig. 3C). First and second free abdominal somites subequal in length, bearing hyaline membrane; hyaline membrane of the first free abdominal somite with serrulate margin, that of the second expanded dorso-medially to form a trapezoidal double-pointed flap, representing a pseudoperculum. Anal somite very short, telescoped within the preceding urosomite (Fig. 3B, D).

Caudal rami (Fig. 3D) subrectangular, ca. 1.8 × as long as wide (measured from base to level of insertion of setae V), with triangular pointed-projection on distal margin dorsally (Fig. 3B); projection 0.4 × as long as ramus length; caudal seta II to VII present, caudal seta I absent; seta II spiniform, with setules along inner margin; seta III plumose, approx. mid-length of seta IV; seta IV shorter than seta V, with breaking planes and plumose; seta V longest, with breaking plane and plumose, sub-equal to urosome length; seta VI slim and plumose. Seta VII inserted dorso-medially near insertion of seta V and seta VI (Fig. 3D). Length ratio of caudal setae to ramus length, from seta II to seta VII: 0.6 : 2.3 : 4.3 : 5.5 : 3.7 : 1.0. Length ratio of caudal setae from seta II to seta VII: 1.0 : 3.7 : 6.9 : 8.9 : 6.0 : 1.5.

Rostrum (Fig. 3E) weakly developed and V-shaped; base broad, completely fused to anterior margin of cephalic shield and tapering to rounded tip between bases of antennules, with two sensillae at middle third of rostrum.

Antennule (Fig. 4A–C) symmetrical, 24-segmented, reaching to distal margin of prosome; ancestral segments II-IV and ancestral segments XXVII–XXVIII completely fused, representing evident segments 2 and 24, respectively. Segments 8 and 9 partly fused, with remnant of ancestral articulation of ancestral segment X and XI, penultimate and ultimate segments sub-equal in length. Armature formula as follows (Roman numeral corresponds to ancestral segment): 1+ae (I), 6+ae (II–IV), 2+ae (V), 2 (VI), 2+ae (VII), 2 (VIII), 2+ae (IX), 2+2ae (X–XI), 1 (XII), 1+ae (XIII), 1+ae (XIV), 1+ae (XV), 1+ae (XVI), 1 (XVII), 1+ae (XVIII), 1 (XIX), 1 (XX), 1+ae (XXI), 1 (XXII), 1 (XXIII), 2 (XXIV), 2+ae (XXV), 2 (XXVI), 5+ae (XXVII–XXVIII).

Antenna (Fig. 4D) biramous. Coxa short, bearing one spinulose seta on distomedial corner. Basis with two sub-equal setae on distomedial corner. Exp 9-segmented, apical segment small, setal formula 1, 1, 1, 1, 1, 1, 1, 1, 3. Endp 2-segmented; proximal segment bearing two setae on medial margin, setae inserted in the same place; distal segment bilobed, bearing three medial setae and six apical setae on medial lobe, with seven apical setae on distal lobe.

Mandible (Fig. 4E) with sclerotised gnathobase comprising ten cuspid or simple teeth and one small dorsal seta on cutting edge of coxal gnathobase. Mandibular palp biramous; basis with four setae on inner margin. Exp 5-segmented, ultimate segment minute, setal formula 1, 1, 1, 1, 2. Endp 2-segmented; proximal segment with four setae on distomedial corner; distal segment with ten apical setae.

Maxillule (Fig. 5A) with praecoxal arthrite bearing nine marginal, spinulose spines and one seta on anterior surface, and four setae on posterior surface. Coxal epipodite with nine apical setae; two proximal ones spinulose, other plumose; coxal endite with four apical setae. Basis fused to exopod, proximal and distal endites armed with four and five apical setae, respectively; basal exite with knob-like appearance and one vestigial seta. Exp with ten setae along apical and outer margin. Endp 3-segmented, proximal and middle segments partly fused, setal formula 4, 4, 7.

Maxilla (Fig. 5B) 6-segmented, comprising praecoxa, coxa, basis and 4-segmented Endp. Praecoxa partly fused to coxa, proximal and distal praecoxal endites with five and three apical setae, respectively. Coxa with two endites, each armed with three apical setae. Basis with large basal endite, armed with four strong apical setae; one of which ornamented with spinule row at mid-length of seta. Endp 4-segmented, setal formula 2, 2, 2, 3; ultimate segment with two long and one short setae.

Maxilliped (Fig. 5C) 8-segmented, comprising syncoxa, basis, and 6-segmented Endp. Syncoxa with four syncoxal endites, setal formula 1, 2, 2, 3; seta on first endite spinulose, basal seta on second endite strong, spinulose; distal endite with one long seta and two short, slender setae. Basis with three medial setae, with row of spinules on anterior surface. Endp with setal formula 2, 4, 4, 3, 3+1, 4; basal seta on first endopodal segment spinulose.

P1–P4 (Fig. 6A–D) biramous, comprising coxa, basis, and 3-segmented rami. Intercoxal sclerite trapezoidal. Coxa rectangular, with seta on distomedial corner. Basis of all swimming legs with lateral seta but lacking in P2; lateral seta inserted on posterior surface. Basis of P1 with robust seta on distomedial corner, with finger-like process on posterior surface arising near base of Exp; process reaching distal margin of Endp-1. Outer distal corner of all endopodal segments drawn out into triangular projection; projection relatively large in P1 and P2. P1Endp-1 without any outer seta. Outer distal corner of P1Exp-2 drawn out into spoon-like process, ornamented with spinules along outer margin. Outer distal corner of Exp-1 and Exp-2 of P2–P4 extended, forming 2-pointed sclerotised expansion, distal pointed process larger than proximal one. Outer spine of Exp-3 of all swimming legs relatively short. P4Exp-3 ca. 3.2 × as long as wide, with elongated, smooth apical spine, as long as segment bearing it and ca. 3 × as long as outer terminal spine, with row of curved spinules at its tip. Armature of swimming legs as presented in Table 1.

**Table 1.** Armament of female thoracic legs P1–P5 in *Boholina laorsriae* sp. nov. (Roman numerals represent number of spines; Arabic numerals represent number of setae).

Swimming leg	Coxa	Basis	Exopod	Endopod
P1	0-1	1-1	I-0; I-1; II, I, 4	0-1; 0-1; 0, I+1, 3
P2	0-1	0-0	I-1; I-1; II, I, 5	0-1; 0-2; 2, 2, 4
P3	0-1	1-0	I-1; I-1; III, I, 5	0-1; 0-2; 2, 2, 4
P4	0-1	1-0	I-1; I-1; III, I, 5	0-1; 0-2; 2, 2, 3
P5	0-1	1-0	I-0; I-1; III, I, 3	0-1; 2, 2, 3

P5 (Fig. 6E) biramous, with 3-segmented Exp and 2-segmented Endp; armament as in Table 1. Coxa and basis as in P3 and P4. Exp-3 ca. twice as long as wide, with apical and outer terminal spines on its tip; apical spine elongated, ca. 1.8 × as long as outer terminal spine, ca. 1.2 × as long as Exp-3 length. Endp much shorter than Exp, reaching level of articulation of Exp-2; Endp-1 as long as wide, without pointed process on outer distal corner; Endp-2 ca.2.6 × as long as wide, with small, pointed process on outer distal corner.

####### Description of adult male.

Body with a total length of 0.65 and 0.67 mm (measured from anterior margin of cephalosome to tip of the projection of caudal rami; mean: 0.66 mm; *N* = 2). Habitus smaller and slenderer than in female (Fig. 7A). Prosome 5-segmented, as in female, ca. 70 % of body length and 2.5 × as long as urosome, with greatest width at posterior end of first pedigerous somite; greatest width ca. 47 % of prosome length. Cephalosome and first three pedigerous somites similar to those in female. Naupliar eye not discernible. Urosome 5-segmented; comprising genital somite, three free abdominal somites and very short anal somite. Genital somite slightly asymmetrical, ca. 25 % of urosome length; posterior margin with hyaline membrane dorsally. First three free abdominal somites similar in length, each with hyaline membrane all around posterior margin; hyaline membrane on third free abdominal somite as in female. Anal somite very short, telescoped within the preceding somite, as in female (Fig. 7B).

Caudal rami (Fig. 7B) relatively shorter than in female, ca. 1.8 × as long as wide. Armament and ornamentation as in female.

Antennule (Fig. 7C–E) asymmetrical. Left antennule non-geniculate, 24-segmented, setal formula as in female. Right antennule geniculate, 22-segmented; armature formula as follows (Roman numeral corresponds to ancestral segment): 1+ae (I), 6+ae (II–IV), 2+ae (V), 2 (VI), 2+ae (VII), 2 (VIII), 2+ae (IX), 1+ae (X), 1+ae (XI), 1(XII), 1+ae (XIII); 1 spiniform seta+ae (XIV), 1+ae (XV), 1+ae (XVI), 1 (XVII), 1 obtuse, fused spine +1+ae (XVIII), 1 obtuse, fused spine +2 (XIX), 1 (XX), 2+ae; one seta spiniform (XXI–XXIII), 4+ae (XXIV–XXV), 2 (XXVI), 5+ae (XXVII–XXVIII).

Antenna, mandible, maxillula, maxilla, maxilliped, and P1−P4 as in female.

P5 (Fig. 8A, B) biramous, asymmetrical. Coxae and intercoxal sclerite fused, forming a common base. Basis rectangular, with outer seta on posterior surface. Left leg biramous, with 3-segmented Exp and 1-segmented Endp; Exp-1 with a long robust outer spine; Exp-2 modified, with a long robust outer spine; Exp-3 highly transformed, bearing several flexible and irregular lobes; outer lobe bearing finger-like appendage; middle lobe prominent, bearing scoop-like appendage; inner lobe, with two elements; innermost (uppermost) one curved, strong seta; other one curved, gutter-like, with serrated concave margin at its cutting edge; Endp flat, oval-shaped, ca. 1.6 × as long as wide. Right leg biramous, with 1-segmented Exp and 1-segmented Endp. Exp with two outer spines, inner spiniform seta, and apical spine, plus spine vestige located subapically on anterior surface; distal outer spine elongated, ca. 3.4 × as long as proximal outer one, ca. 2.7 × as long as apical spine; subapical and apical spines machete-shaped, subapical spine vestige ca. 0.7 × as long as apical spine; apical spine ca. 0.4 × as long as distal outer spine; inner spiniform seta minute, located at level of insertion of proximal outer spine; Endp as long as Exp, ca. 3 × as long as wide, armed with two sub-equal spines.

####### Differential diagnosis.

The new species was confidently identified to the genus *Boholina* based on the combination of the following characteristics mentioned by Fosshagen and Iliffe (1989):

(1) fourth and fifth pedigerous somites fused,

(2) rostrum with a rounded tip,

(3) genital pores paired and separate,

(4) caudal rami with cuticular pointed projection distally,

(5) distal outer corner of all endopodal segments of P1 forming a triangular pointed projection,

(6) P1Endp-3 without outer seta,

(7) P4Exp-3 with modified apical spine,

(8) female P5 with a 2-segmented Endp,

(9) male left P5Exp-3 modified to unique characteristic grasping organ, and

(10) female and male with 4- and 5-segmented urosome, respectively; anal somite very short and sometimes concealed within the preceding somite.

Examination of the structure of the genital double-somite, of P4 and P5 in both males and females, the relative length of subapical spine vestige on the male right P5 and the shape of the male left P5Endp revealed that the new species is most similar to *B. crassicephala*, which had previously been described from a pool in a cave of Bohol Island, the Philippines. Several characters are shared by both species, especially the structure of the male P5. However, there are also remarkable differences (Table 2). The characteristics which obviously distinguish *Boholina laorsriae* sp. nov. from *B. crassicephala* are as follows:

**Table 2.** Morphological comparison of the six species of genus *Boholina.* Abbreviations: GDS, genital double-somite; CR, caudal rami; A2, antenna; Mb, mandible; Mx, maxillule (*measured from figures of the original description).

**Character**	***B. ganghwaensis***	***B. parapurgata***	***B. munaensis***	***B. purgata***	***B. crassicephala***	***B. laorsriae* sp. nov.**
**Female**						
Body length (mm)	1.03–1.29	0.93–1.11	0.70–0.77	0.73–0.79	0.75–0.85	0.68–0.73
Posterior lateral corner of second and third pedigerous somites	Pointed	Pointed	Rounded	Pointed	Rounded	Rounded
Genital pores on GDS	Either side of ventralmidline	Either side of ventralmidline	Ventrolaterally	Either side of ventralmidline	Ventrolaterally	Ventrolaterally
Hook–like process on genital pore plate	Yes	No	No	No	No	No
Length/width ratio of CR	1.6	1.5	1.5	1.6*	1.8*	1.8
Distal two segments of A2 Exp	Separated	Separated	Separated	Fused	Fused?	Separated
Number of setae on distal endopodal segment of Mb	11	10	10	10	10	10
Setal formula of Mb Exp	1,1,1,1,2	0.1,1,1,2	0,1,1,1,2	1,1,1,1,2	1,1,1,1,2	1,1,1,1,2
Basis and first endopodal segment of Mx	Partly fused	Fused	Fused	Fused	Fused	Separated
Length ratio of apical and outer terminal spines of P4Exp–3	1.6*	1.5	1.9	1.5	2.5	3.0
Length/width ratio of distal endopodal segment of P5	2.5	2.6	2.6	1.8*	1.6*	2.6
Length ratio of apical and outer terminal spine of P5Exp–3	1.0	0.8	1.4	0.8*	1.2*	1.8
Length ratio of apical spineand P5Exp–3	0.9	0.9	0.8	0.8*	1.0*	1.2
**Male**						
Body length (mm)	0.87–0.93	0.66–0.71	0.68	0.64–0.73	0.70–0.77	0.65–0.67
Length ratio of left and right Endp of P5	1.1*	0.8*	0.5	0.7*	0.8*	0.45
Length/width ratio of right P5Endp	3.2	3.6	3.5*	2.6*	2.7*	3.0
Right P5Endp with large inner spiniform process	No	No	Yes	No	No	No
Right P5Endp armature	2 slender spines	2 sigmoid spines	Absent	2 slender spines	2 slender spines	2 slender spines
Length ratio of two outer exopodal spines on right P5	1.5	1.8	1.3	1.7*	1.7*	3.4
Relative length of subapical spine compared to apical spine on left P5Exp	Less than 0.5 ×	Less than 0.5 ×	More than 0.5 ×	Less than 0.5 ×	More than 0.5 ×	More than 0.5 ×

i) Apical spine of the female P5Exp-3 is ca. 1.8 × as long as outer terminal spine in the new species, but it is sub-equal to the outer terminal spine found in *B. crassicephala*.

ii) Exopodal segment of the male right P5 has medial minute seta in the new species; however, it is absent in *B. crassicephala*.

iii) Distal outer spine on exopodal segment of the male right P5 is relatively long, and the distal outer spine is ca. 2.9 × as long as the proximal one; however, in *B. crassicephala*, the spine on exopodal segment of the male right P5 is relative shorter and the distal outer spine is ca. 1.9 × as long as the proximal one.

iv) The male left P5Endp is relatively smaller in the new species than that of *B. crassicephala*.

The Thai *Boholina* can be easily distinguished from *B. purgata*, *B. parapurgata*, and *B. ganghwaensis* by the characteristics of the widely separated genital pores, relatively longer subapical spine vestige on the male right P5 when compared to the length of apical spine, the higher length ratio of the apical spine to the outer terminal spine in the female P5Exp-3 and the elongated apical spine of the female P4Exp (Table 2). The genital double-somite of the new species is barrel-shaped, while it is globular in *B. munaensis*. Additionally, the ii) characteristic is unique for the Thai *Boholina*. Based on the characteristics used in Moon and Soh (2014) accompanied by additional ones, the morphological characteristics of the six species are presented in Table 2.

####### Remarks.

Only six specimens were collected from a pool and the new species was not encountered in the other eight caves visited in this research project. Freshwater Cyclopoida belonging to the genera *Thermocyclops, Metacyclops*, and *Mesocyclops*, as well as harpacticoids of the genus *Schizopera* and of the family Ectosomatidae were also collected from the type locality.

### Key to the adult of *Boholina*

Boxshall and Jaume (2012) provided key to adults of the genus based on the four described species. In this paper, two more species from Korea and Thailand are added in the key.

**Table:** 

1	Female genital double-somite globular-shaped, as long as wide; male right P5Endp unarmed, with large inner spinous process	***B. munaensis* Boxshall & Jaume, 2012**
–	Female genital double-somite barrel-shaped, longer than wide; male right P5Endp armed with 2 elements, without large inner spinous process	**2**
2	Female genital pores located ventrolaterally; postero-lateral corners of second and third pedigerous somites rounded in both sexes; male right P5Exp with relatively large spine vestige; spine vestige longer than half length of apical spine	**3**
–	Female genital pores located close together on either side of body midline; postero-lateral corners of second and third pedigerous somites pointed in both sexes; male right P5Exp with relatively small spine vestige; spine vestige shorter than half length of apical spine	**4**
3	Apical spines on female P5 sub-equal in length; male right P5Exp without inner spiniform seta; male left P5 Enp large, as long as right P5Endp	***B. crassicephala* Fosshagen & Illife, 1989**
–	Inner apical spines on female P5 ca. 1.6 × as long as outer one; male right P5Exp with inner spiniform seta; male left P5 Enp small, much shorter than right P5Endp	***B. laorsriae* sp. nov.**
4	Apical spine on female P5Exp-3 longer than outer terminal spine; gonoporal plate without small hook-like process	**5**
–	Apical spine on female P5Exp-3 slightly shorter than outer terminal spine (0.96 x); gonoporal plate with small hook-like process	***B. ganghwaensis* Moon & Ho, 2014**
5	Male right P5Endp ca. 2.6 × as long as wide, bearing two slender spines; distal endopodal segment of female P5Endp ca. 1.8 × as long as wide; outer terminal spine on female P5Exp-3 shorter than segment bearing it	***B. purgata* Fosshagen & Illife, 1989**
–	Male right P5Endp ca. 3.6 × as long as wide, bearing two sigmoid spines; distal endopodal segment of female P5Endp ca. 2.6 × as long as wide; outer terminal spine on female P5Exp-3 longer than segment bearing it	***B. parapurgata* Boxshall & Jaume, 2012**

## Discussion

Calanoid copepods are ubiquitous in marine, brackish, and fresh waters, comprising 44 families and approximately 330 genera (Walter and Boxshall 2019). However, compared to the diversity of cave-dwelling Cyclopoida and Harpacticoida, stygobiotic Calanoida is less diverse in terms of numbers of species. In Southeast Asia, seven stygobiotic calanoid species have been previously recorded from caves in Vietnam, the Philippines, and Indonesia, including *Boholina* (four species, excluding the new species described here), *Hadodiaptomus* Brancelj, 2005 (one species), and *Nannodiaptomus* Dang & Ho, 2001 (two species) (Brancelj 2005; Boxshall and Jaume 2012; Tran and Brancelj 2017). Of these, only representatives of *Boholina* were described from anchialine caves.

The genus *Boholina* clearly shows affinities to the families Ridgewayiidae and Pseudocyclopidae, by the presence of several plesiomorphic characters in the antennule, antenna, mouthparts, and swimming legs. Nearly all species of *Boholina* were collected from anchialine caves: this habitat is different from a benthic environment in shallow water where the two families have frequently been found (Fosshagen and Iliffe 1989). Recently, the families Boholinidae and Ridgewayiidae were synonymised in the family Pseudocyclopidae, and the genus *Boholina* is sister to the clade of the genera *Ridgewayia, Stygoridgewayia, Hondurella, Placocalanus*, and *Pseudocyclops*, based on a morphology-based cladistic analysis of Bradford-Grieve et al. (2014). From a geographical point of view, *Boholina* has been recorded only from East and Southeast Asian countries along the western coast of the Pacific Ocean (Fig. 1). This suggests an Asian origin of the genus. The assumption is supported by the fact that its most closely related genera (*Ridgewayia*, *Stygoridgewayia*, *Placocalanus*, and *Pseudocyclops*) can also be found in this region. The only genus that has yet to be found in Asia is *Hondurella*, which was only obtained from Utila Island of Honduras in the Caribbean basin (Suárez-Morales and Iliffe 2007).

The geographical distribution of the new species is slightly different from those of all other species of *Boholina*, as it was collected from a freshwater pool within a cave located very far from the sea (ca. 6.5 km) compared with those of *B. crassicephala* and *B. purgata* (200 m), *B. parapurgata* and *B. munaensis* (700 m), and *B. ganghwaensis* (inter-tidal mudflat). The occurrence of the new species in a cave located so far from the coast is the same as that of *Stygoridgewayia*, which were collected from bores/wells located up to 450 km inland from the coast in the Cape Range Peninsula and Pilbara region, Western Australia (Tang et al. 2008). Tang et al. (2008) hypothesised that the occurrence of *Stygoridgewayia* in subterranean waters is due to secondary colonisation of freshwater after the regression of the epicontinental sea which inundated a large part of the land. From this geographical viewpoint, we postulate that the ancestor of the present-day population of Thai *Boholina* could have penetrated the cave in either the Cretaceous or Miocene periods. The assumption was postulated as the geological evidence shows that the sedimentary rocks in the area of the present-day Satun province and north-western Malay Peninsula were formed under the ancient sea for a very long time, spanning the Late Cambrian and Triassic periods, before uplifting of the area in Cretaceous, and the Quaternary sediments in the area below 10 m a.s.l. were interpreted as deposits of the epicontinental sea in the Holocene (The Malaysian and Thai Working Groups 2009).

Based on structure (i.e., degree of modification) of mouthparts, especially with respect to the mandible, maxilla, and maxilliped, feeding habits were suggested for several taxa of the families Epacteriscidae, Ridgewayiidae, and Pseudocyclopidae (e.g., Fosshagen 1968; Fosshagen et al. 2001; Jaume and Humphreys 2001; Fosshagen and Iliffe 2003, 2004a, 2004b, 2007; Suárez-Morales and Iliffe 2007). The absence of the conspicuous modification of the raptorial feeding habit in the mandible, maxilla, and maxilliped in Thai *Boholina* and its congeners suggests that they are particle feeders. The characters that indicate particle-feeding habits in *Boholina* and in several ridgewayiids, such as *Ridgewayia*, *Brattstromia*, *Exumellina*, and *Stargatia* are are as follows: 1) the mandible bears numerous small teeth on the cutting-edge of the gnathobase and the endopod is well-developed, with four and more than nine setae on proximal and distal segments, respectively, and 2) the maxilla and maxilliped are armed with normal plumose setae. In the raptorial feeders, like epacteriscids and some ridgewayiids, such as *Exumella, Palmeriella*, and *Normancavia*, the general modifications of these three appendages include: 1) an enlargement of the teeth, especially the ventralmost teeth, and the reduction of the endopod, and 2) the transformation of setae on the distal part of the maxilla and maxilliped to stout, elongate, spinous setae or the reduction of setae on both the maxilla and maxilliped.

Even if many calanoid taxa in the superfamily Pseudocyclopoidea Giesbrecht, 1893 were collected from anchialine caves, it is likely that there is no specific adaption/modification in relation to the cave habitat in this family. Such a morphological adaptation or modification generally corresponds to the zones of the water column in which the copepod lives. Based on the relative length of the antennule, we suggest that the genus *Boholina* is a hyperbenthic form, because it has relatively short antennules (not extending beyond prosome) as in most epacteriscids. In the genera *Exumellina* and *Stargatia*, which were collected in the water column of anchialine caves, the antennules extend beyond the prosome (Fosshagen and Iliffe, 1998, 2003). In our opinion, only the reductions of the eyes and outer seta on P1Endp are adaptations of *Boholina* corresponding with life in caves, as Brancelj and Dumont (2007) suggested for the freshwater stygobiotic Calanoida.

## Supplementary Material

XML Treatment for Boholina laorsriae
